# Supplementary material for: Combined docking and machine learning identify key molecular determinants of ligand pharmacological activity on β2 adrenoceptor
Source: Pharmacol Res Perspect. 2022 Aug 26;10(5):e00994. doi: 10.1002/prp2.994 (PMC9418666; doi:10.1002/prp2.994)
Supplement: Supplementary file 1 — Figure S1–S6 [file PRP2-10-e00994-s002.pdf]

Supplementary data

Supplementary Figure 1

A

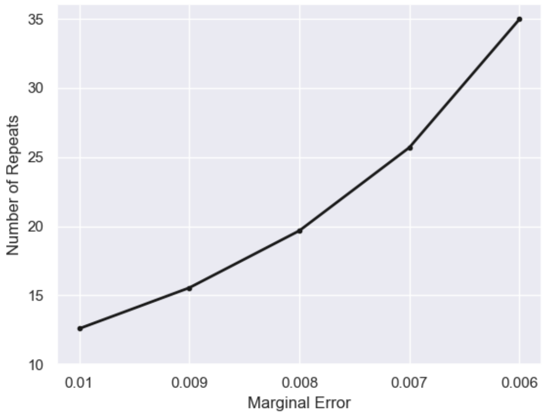

B

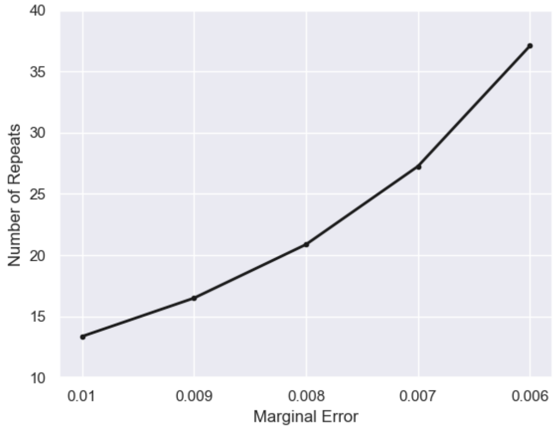

Supplementary Figure 1. Determining the optimal number of repeats required to estimate, with 99% confidence, true model performance to a specified level of precision. (A) RFC model on the filtered dataset and (B) XGBoost model on the unfiltered dataset. In this case, 13 repeats were sufficient for both models.

Supplementary Figure 2

A. FILTERED DATASET

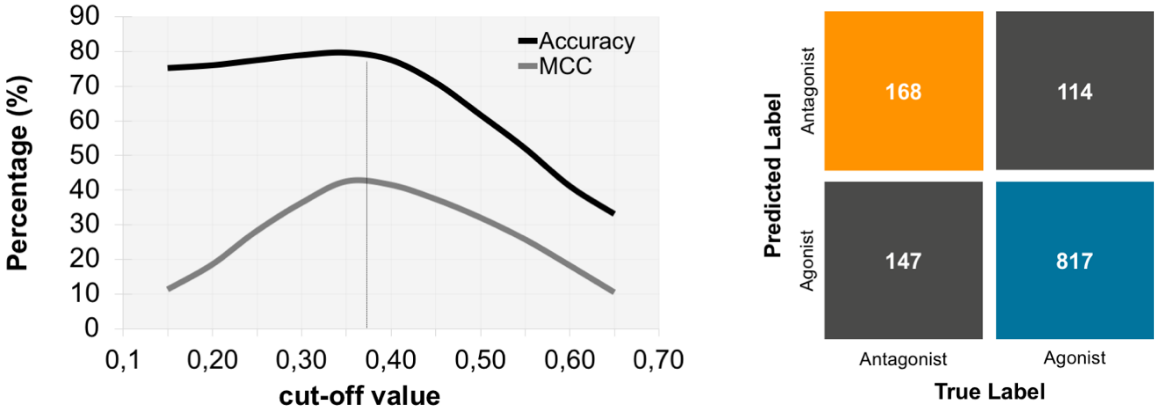

B. FULL DATASET

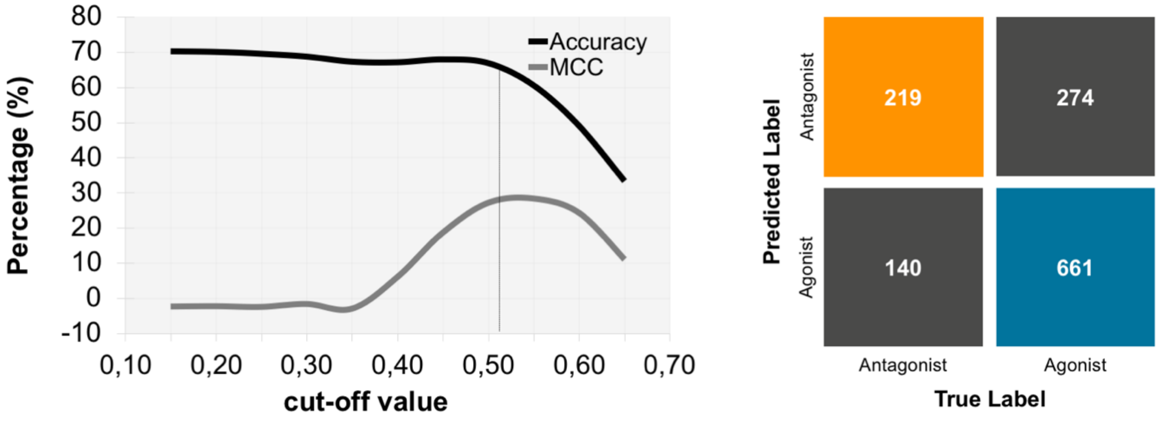

Supplementary Figure 2. Cut-off graph to obtain the maximum MCC value for the Pearson's correlation approach and the corresponding confusion matrix to differentiate agonist and antagonist. (A) Filtered dataset, right: cut-off and left: confusion matrix MCC = 0.37. (B) Full dataset, right: cut-off and left: confusion matrix MCC = 0.51.

Supplementary Figure 3

FILTERED DATASET

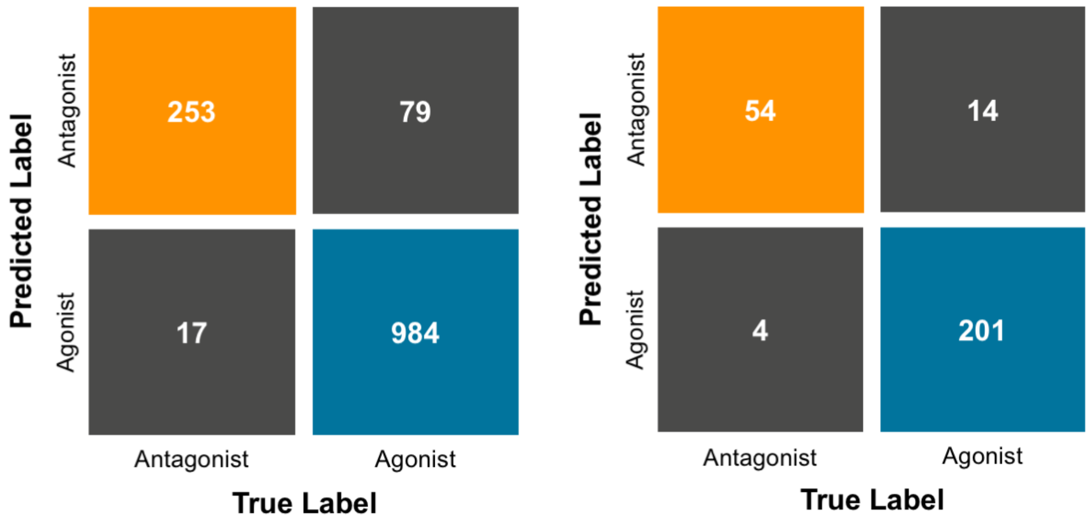

Supplementary Figure 3. Random Forest Classification Confusion Matrix for Cross-Validation (left) and Hold-out sets (right) for the filtered dataset. The left figure shows the median confusion matrix across all K-folds and repeats, the right shows the hold-out set. Y-axis shows the frequency of classes predicted by the model, across all samples. The x-axis shows the true known class label. Overall the confusion matrix provides insight into the ratios of false positive & negatives made by the model across samples for the filtered dataset.

Supplementary Figure 4

FULL DATASET

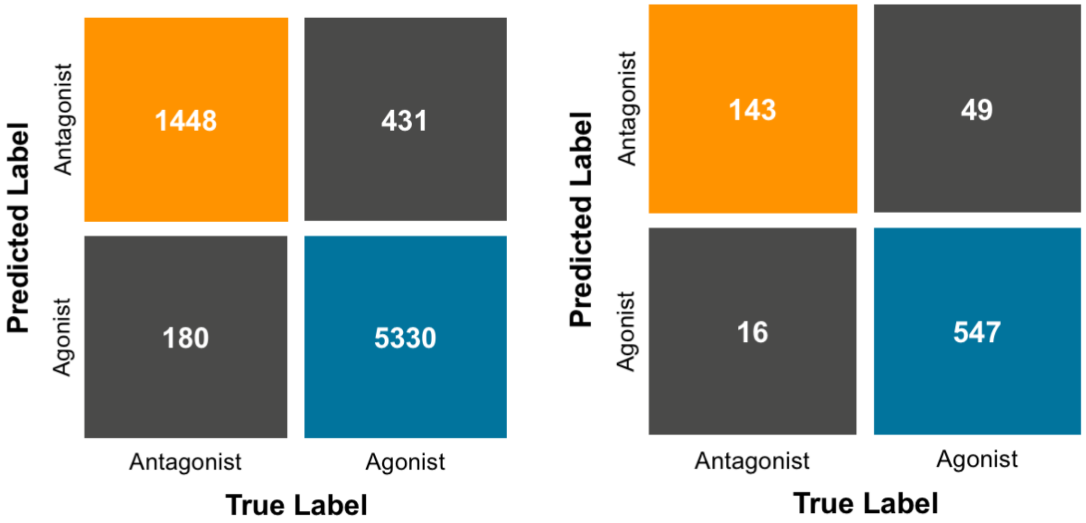

Supplementary Figure 4. XGBoost Confusion Matrix for Cross-Validation (left) and Hold-out sets (right) for the full dataset.

Supplementary Figure 5

A) FILTERED DATASET

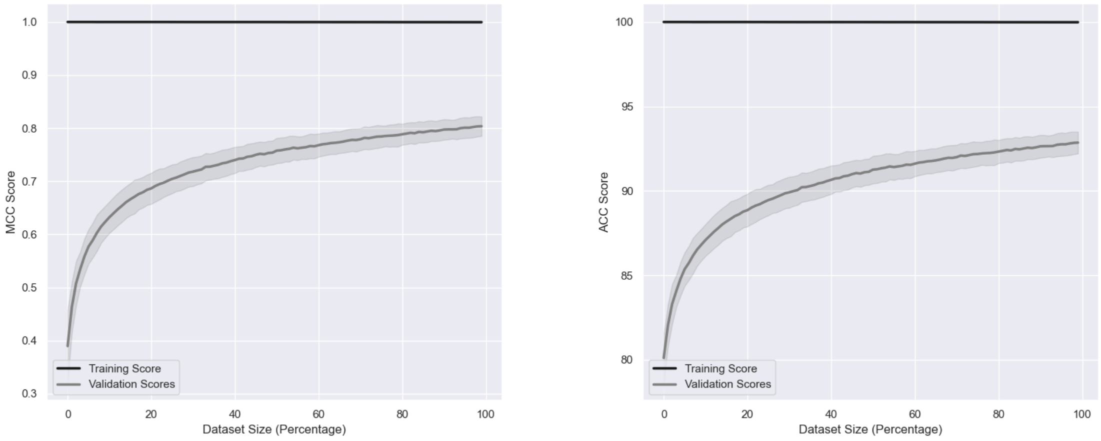

B) FULL DATASET

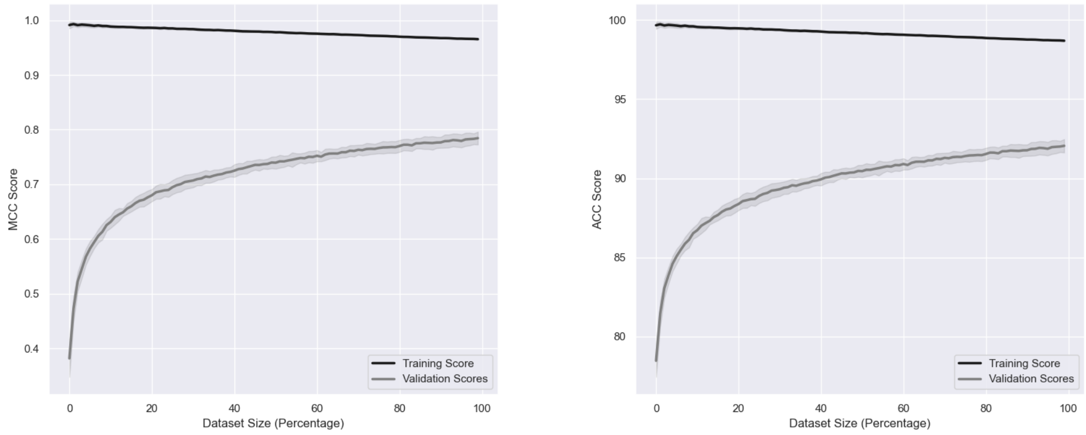

Supplementary Figure 5. Learning curves for the MCC Scores (left) and accuracy – ACC Scores (rights) for the (A) RFC model on the filtered dataset and the (B) XGBoost model on the unfiltered dataset.

Supplementary Figure 6

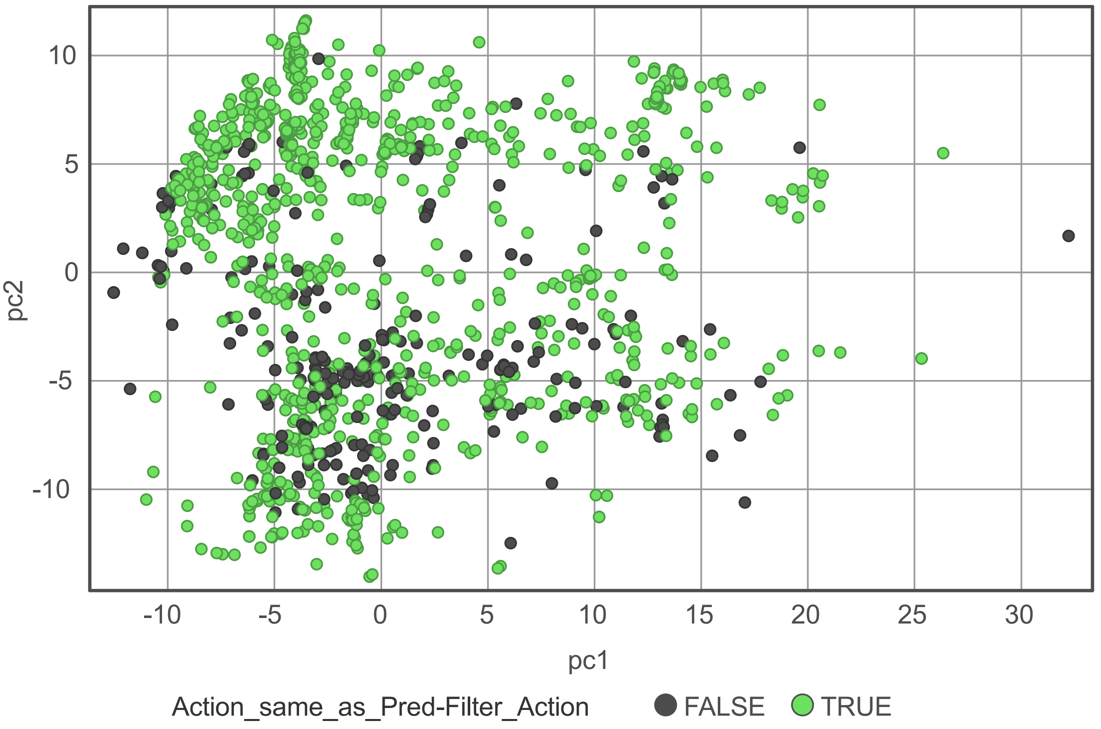

Supplementary Figure 6. Principal Component Analysis (PCA) biplot of the filtered dataset. In grey (as in Supplementary Figures 2-4), the false predicted ligands which include the antagonist predicted agonist and the agonist predicted antagonist. In green, the true predicted ligands.

Additional files:

Supplementary Tables 1-4 are in included as an Excel file “prp2994-sup-0002-TablesS1-4.xlsx”.
